# Endometrial Stromal Sarcomas: A clinico-pathological analysis of 27 patients

**DOI:** 10.12669/pjms.291.2235

**Published:** 2013

**Authors:** Dae-Young Kim, Kyung-Taek Lim, Yong-Soon Kwon

**Affiliations:** 1 Dr. Dae-Young Kim, Departments of Anesthesiology and Pain Management, Ulsan University Hospital, Collage of Medicine, Ulsan University, Ulsan, Korea.; 2 Dr. Kyung-Taek Lim, Department of Obstetrics and Gynecology, Ulsan University Hospital, University of Ulsan College of Medicine, Dong-gu Ulsan, Korea; 3 Dr. Yong-Soon Kwon, Department of Obstetrics and Gynecology, Ulsan University Hospital, University of Ulsan College of Medicine, Dong-gu Ulsan, Korea

**Keywords:** Endometrial stromal sarcoma, Clinical and pathological features, Rare tumor

## Abstract

***Objective:*** To evaluate clinico-pathological features and prognostic valuses of Endometrial stromal sarcomas (ESS) through comparison of the two grade groups (low- and high-grade disease).

***Methodology: ***We retrospectively analyzed the medical records of 27 patients who were diagnosed with ESS at a single institute between March 1988 and November 2009. Our retrospective chart review was approved by our local institutional Review Board (IRB).

***Results:*** The median age of the patients was 44.0 years, the median follow-up period was 101.0 months and the 10-year survival rate was 74.2%. The median uterine weight was 215.0 gm. Twenty-three (70.4%) and four patients (29.6%) had low- and high-grade disease, respectively. As primary treatment, twenty-four (70.4%) and three patients (11.1%) underwent type I hysterectomy and type III hysterectomy, respectively. Total six cases were recurred and two cases of the six-recurred patients were distant metastasis (lung) and four cases were died of the disease. Univariate analysis revealed that the histologic grade and the uterine tumor weight were significantly related with longer disease-free survival (p=0.025 and 0.043 respectively).

***Conclusion: ***ESSs with high-grade or larger tumor size have to be carefully and sufficiently managed, because of its rarity and aggressive behavior. To determine the proper adjuvant treatment of ESS with high risks, further clinical data should be collected and studied.

## Introduction

 Endometrial stromal sarcomas (ESSs) are rare tumors, constituting about 2% to 4% of all uterine malignant tumors.^1^^-^^3^ Because of its rarity and preoperative benign-looking appearance such as a uterine myoma, a preoperative diagnosis is difficult. Accurate diagnosis was made by intra-operatively frozen biopsy or permanent pathologic report, which showed ESSs.

 The classification and nomenclature of these neoplasms have evolved since they were first conceived by Norris and Taylor in 1966.^4^ Currently, the 2003 World Health Organization (WHO) classification divides endometrial stromal tumors (EST) into 3 different subsets^2^:

1. Endometrial stromal nodule (ESN)

2. Low-grade endometrial stromal sarcoma (ESS)

3. Undifferentiated endometrial sarcoma (UES)

 ESN and low-grade ESS are composed of cells resembling endometrial proliferative stroma with a plexiform vascular arrangement and minimal cytological atypia. UES is characterized by marked cellular pleomorphism, high mitotic index, and frequent presence of necrosis; usually it has an aggressive clinical behavior, with metastases and bad prognosis, at variance with LG-ESS. There are still controversies in the classification criteria of ESS.

 The standard surgical treatment is still controversial. In patient without a desire of fertility or with menopause, a total hysterectomy and bilateral salpingo-oophorectomy was recommended. However, Li et al^5^ recently demonstrated that ovarian preservation could be a safe option for surgical treatment in stage I, low-grade ESS. The importance of surgical staging operation and adjuvant treatment is still unknown.^6^^,^^7^

 The present study was aimed to evaluate the clinico-pathologic features and the prognostic values of each the two grade groups ESSs, which could make a help determine a proper management strategy of each grade ESSs.

## Methodology

 This study included 27 patients with the pathologically diagnosed ESS treated between March 1988 and November 2009 in the Cheil General Hospital and Women’s Healthcare Center, Seoul, Korea.

 Retrospectively, medical records were analyzed for information on the demographic characteristics, surgical findings, pathologic findings, and clinical outcomes of follow-up.

 The files and material from all the patients were reevaluated, including demographic (age), clinical (symptoms, parity, menopause, and treatment), image, surgical, staging (International Federation of Gynecology and Obstetrics [FIGO]), and follow-up data at November 2010. All eligible 27 patients’ specimens were reviewed and confirmed pathologically as ESSs by our department of pathology.

 The surgical treatments were subdivided into total abdominal hysterectomy (TAH), laparoscopic assisted vaginal hysterectomy (LAVH) or vaginal hysterectomy (VH), and radical abdominal hysterectomy (RAH). Adjuvant therapy included radiotherapy, hormonal therapy, and chemotherapy. The chemotherapeutic regimens were not standardized; however, the main regimen consisted of ifosfamide, Adriamycin, and platinum based-agents. The twenty seven patients were divided in two groups: low-grade ESS and high-grade ESS.

 To compare the features of the 2 tumor groups, χ2 Test, Fisher test, and Mann–Whitney test were used. Kaplan-Meier curves were used to calculate the mean DFS and OS, and the log-rank test was applied for univariate analysis (histological type, age, menopause, size, mitotic index, and necrosis). The statistical Package for social Science (SPSS, Inc., Chicago IL) was used for the statistical analysis. The differences were considered significant at a level of P<.05.

## Results

 The median age of entire population was 44.0 years (range, 20-79). The median follow-up period was 101.0 months (range, 10-206). The detail demographics of 27 patients are shown in Table-I. Most patients were premenopausal (81.5%) and the main symptoms were abnormal uterine bleeding (59.3%) and palpable mass (25.9%). 

**Table-I:** Characteristics of patients.

	*Patients (n=27)*
Median age (years)	44.0 (range, 20-79)
Median BMI	21.5 (range, 18.8-32.9)
*Menopause*	
Yes	5 (18.5%)
No	22 (81.5%)
*Diagnostic specimen*	
curettage specimens	5 (18.5 %)
hysterectomy specimen	22 (81.5%)
*Main symptoms*	
Vaginal bleeding	16 (59.3%)
Palpable mass	7 (25.9%)
Others *Stage* I III IV Unknown	4 (14.8%) 17 (62.9%) 3 (11.1%) 4 (14.8%) 3 (11.1%)

**Table-II:** Operative and pathologic findings

	*Patients (n=27)*
Uterine weight (gm)	Median weight, 215.0 (range, 80-778)
*Operation type*	
TAH	19 (70.4%)
LAVH or VH	5 (18.5%)
RAH	3 (11.1%)
*Grade*	
Low	23 (70.4%)
High	4 (29.6%)
*Depth of myometrial invasion*	
Less than half myometrium	14 (51.9%)
More than half myometrium	13 (48.1%)
*LVSI*	
Yes	15 (55.6%)
No	12 (44.4%)

TAH: Total abdominal hysterectomy.

LAVH: Laparoscopic assisted vaginal hysterectomy.

VH: Vaginal hysterectomy. RAH: Radical abdominal hysterectomy. LVSI: Lymphovascular space invasion

 The surgical and pathological findings are summarized in Table-II. The median tumor’s weight was 215.0gm (range. 80-778). All patients underwent surgical treatment with or without adjuvant therapy. Type I hysterectomy was performed in 24 patients (88.8%) and type III hysterectomy in three patients (11.1%). The distribution of the deep myometrial invasion and lymphovascular space invasion (LVSI) were even, respectively. High-grade ESSs were identified in four patients (29.6%).

Table-III showed the comparison between patients with adjuvant therapy and patients without adjuvant therapy. The factors affecting the decision to do adjuvant therapy were high-grade (100%) and advanced FIGO stage 6 / 7 (85.7%) patients with advanced disease. 

 The recurrence rate was 6/ 27 patients (22.2%) and in patients with high-ESSs, 2/4 (50%) patients were recurred. Based on univariate analysis, grade and tumor weight were significant prognostic factors, which were affecting Disease-Free Survival (DFS), respectively (p value: 0.004, 0.025) in Table-IV.

**Fig.1:**
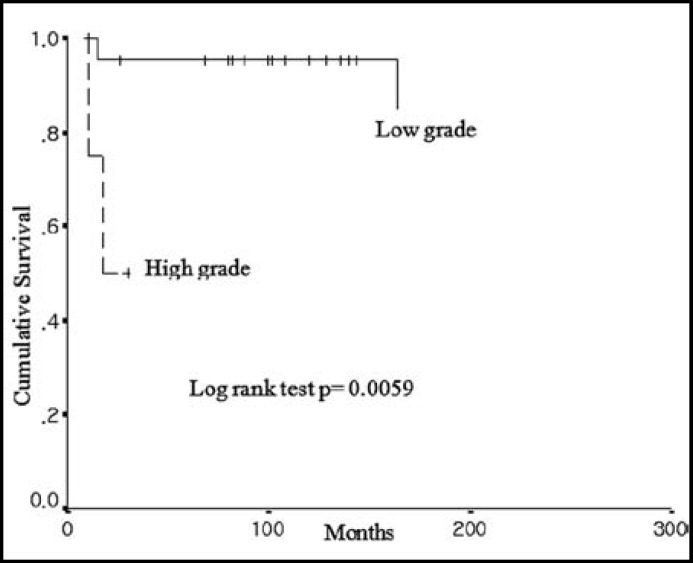
Overall survival as a function of ESS grade

**Fig.2:**
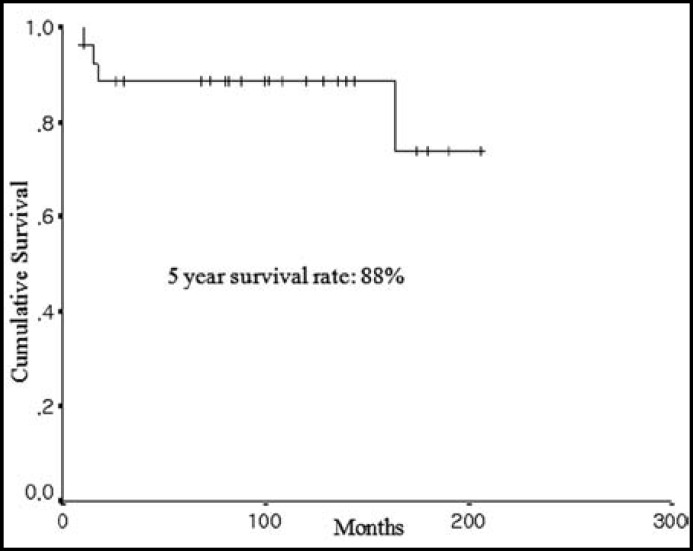
Overall survival of 27 patients with ESS

 Five year survival rate was 88.6% and 10-year survival rate was 74.2% (Fig.1). Four patients in total six patients with recurrence were died. The mean survival was 191 months (95% Confidence Interval: 171-211) in patients with low-grade ESSs and was 43 months (95% Confidence Interval: 14-73) in patients with high-grade ESSs. Overall survival rate was significantly lowered in high-grade ESSs rather than low-grade ESSs (p value = 0.0059) (Fig.2).

## Discussion

 Up to date, most gynecologic tumors have been discovered earlier in clinical stage with the help of routine check-up and increasing of patient’s insight for healthy life. Except advanced stages, uterine tumors with early stage are asymptomatic and mimics clinical nature of benign uterine tumor, such as uterine leiomyoma. These findings make it difficult to be definitely diagnosed from other diseases preoperatively. 

 Consistent with previous reports^8^^,^^9^, in our study, the definitive diagnosis was made mainly on hysterectomy specimen rather than on curettage specimen (only five cases on curettage specimens and the others, 22 cases, made on hysterectomy specimen) (Table-I).

**Table-III:** Comparison between patients with adjuvant therapy and patients without adjuvant therapy

	*Adjuvant therapy* * (n=16)*	*No adjuvant therapy* *(n=11)*	*P value*
Stage			0.362
I	10 (62.5%)	10 (90.9%)	
III	3 (18.8%)	0	
IV	3 (18.8%)	1 (9.1%)	
Grade			0.123
Low	12 (75.0%)	11 (100.0%)	
High	4 (25.0%)	0	
Depth of myometrial invasion			0.440
Less than half myometrium	7 (43.8%)	7 (63.6%)	
More than half myometrium	9 (56.2%)	4 (36.4%)	
LVSI			
Yes	12 (75.0%)	3 (27.3%)	0.022
No Adjuvant therapy RTx CTx HTx.	4 (25.0%) 2 (12.5%) 5 (31.3%) 9 (56.3%)	8 (72.7%)	

LVSI: Lymphovascular space invasion. RTx: Radiotherapy, CTx: Chemotherapy, HTx: Hormonal therapy

**Table-IV:** Clinical outcomes of patients

	*Patients with recurrence*	*Patients without recurrence*	*P value*
	*(n= 6)*	*(n=21)*	
Grade			
Low	4	19	0.004*
High	2	2	
Adjuvant therapy			
Yes	6	10	0.336
No	0	11	
Uterine weight (gm)	418.8±171.3	228.9±156.4	0.025*
Depth of myometrial invasion			
Less than half myometrium	3	11	0.847
More than half myometrium	3	10	
LVSI			
Yes	5	10	0.564
No	1	11	

LVSI: Lymphovascular space invasion.

*: P value < 0.05

 In the twenty-two case, the majority of preoperative diagnosis was uterine leiomyoma without any suspicion of ESS. These results might be confusing the establishment of definitive guideline for surgical treatment.

 Low-grade ESS is treated surgically, primarily by total hysterectomy and bilateral salpingo-oophorectomy. The role of comprehensive surgical staging, including pelvic and para-aortic lymphadenectomy remains controversial. Adjuncts in treatment include chemotherapy, radiotherapy, and/or hormonal therapy including aromatase inhibitors, gonadotrophin-releasing hormone (GnRH) analogs, and progestins.^10^^,^^11^

 The standard guidelines of ESS treatment have not been agreed upon. The initial surgical treatment strategies are important for improving the survival outcomes.^6^^,^^12^ However, the extent of surgical treatment is controversial. Similar to previous report^9^, our study showed that one patient who underwent lymphadectomy for low-grade ESS had lymph node metastasis in the pelvic area. However, in this case, the weight of specimen was 778gm and the depth of myometrium was full thickness. In other words, small sized ESSs with early clinical stage have to be evaluated for the significance and need for lymphadectomy. Up to date, the therapeutic benefit of radical lymphadenectomy is still uncertain.

 Low-grade ESS is an indolent tumor and the estimated overall survival ranges from 69% to 84% at 5 years, from 65% to 76% at 10 years of age.^4^^,^^13^ Recurrence occurs in up to half of the patients, sometimes many years after the initial diagnosis. The most frequent sites of recurrence include pelvis and abdomen.^2^^,^^14^ In the present study, 5 year survival rate was 88.6% and 10-year survival rate was 74.2 % (Fig.1). These survival results were comparable with the previous studies because our study included total ESSs, which included high-grade ESSs. The recurrence rate was 22.2% (6/ 27 patients). Pure recurrence rate of low-grade ESSs was 17.4% (4/23 patients). Four patients in total six patients with recurrence were died.

 Although ESSs have a relatively good survival outcomes, low recurrence rate, and longer time of recurrence, Adjuvant therapeutic strategy should be needed after primary surgical treatment was performed. However, there are several limitations in undertaking adjuvant therapy.

 The previous report^9^ showed that Adjuvant therapy, regardless of the treatment modality, should be taken to improve the survival outcomes. However, our study revealed some different data compared to the report by Nam EJ et al.^9^ In our study, total eleven patients did not receive any adjuvant therapy and all these patients have no recurrence (Table-IV). This point is very important because these results showed that we have to consider the selection of patients who need any adjuvant therapy or not. To stratify ESSs for determining the indication of adjuvant therapy, we need larger number of patients with ESSs and the values of the prognostic factors.

 Relatively well known prognostic factors included stage, tumor size, lymphovascular space invasion (LVSI), mitotic index, and nuclear atypia. In our study, these factors were evaluated as significant prognostic factor affecting on the survival outcomes. Fig.1 shows that the overall survival outcomes were better in low-grad ESSs than in high-grade ESSs (p value = 0.0059). Also, tumor size (weight of tumor) had a significantly prognostic value. Larger tumor size of ESSs had poorer overall survival outcomes. Other factors did not showed the significant values in recurrence rate, which included depth of myometrial invasion, LVSI, and adjuvant therapy (Table-IV).

 In patients with ESSs, to obtain any survival benefits and prevent tumor recurrence, we have to evaluate each prognostic factor in large number study and the need of adjuvant therapy in the selected group to avoid unnecessary adjuvant therapy. Also, if there is the consensus of adjuvant therapy, we have to study which modality of adjuvant therapy is the best effective.

 Similar to the previous studies, our study had small number of patients to receive adjuvant therapy. Because of this small number, it is difficult to obtain the statistically powerful data, which evaluate the survival benefits and the toxicities of each modality of adjuvant therapy (chemotherapy, radiotherapy, and/or hormonal therapy).

 In summary, our experience for ESSs demonstrated that the tumor size and the grade were important factors related to progression free survival. However, because of the small cohort arising from the rarity of this tumor, the statistical analysis is limited to value. Therefore, further studies are warranted to determine the definite principle of management through multicenter using randomized clinical trial.
